# Trends in drug development for rare and intractable diseases based on the KEGG NETWORK

**DOI:** 10.1093/narmme/ugae009

**Published:** 2024-08-09

**Authors:** Mao Tanabe, Makoto Hirata, Ryuichi Sakate

**Affiliations:** Laboratory of Rare Disease Information and Resource Library, Center for Intractable Diseases and ImmunoGenomics (CiDIG), National Institutes of Biomedical Innovation, Health and Nutrition (NIBIOHN), Ibaraki, Osaka 567-0085, Japan; Laboratory of Rare Disease Information and Resource Library, Center for Intractable Diseases and ImmunoGenomics (CiDIG), National Institutes of Biomedical Innovation, Health and Nutrition (NIBIOHN), Ibaraki, Osaka 567-0085, Japan; Laboratory of Rare Disease Information and Resource Library, Center for Intractable Diseases and ImmunoGenomics (CiDIG), National Institutes of Biomedical Innovation, Health and Nutrition (NIBIOHN), Ibaraki, Osaka 567-0085, Japan

## Abstract

The pathophysiological mechanisms underlying many rare and intractable diseases remain unclear, and there are few drugs for the treatment of these diseases. An understanding of approved drugs is important to improve drug development. In DDrare (Database of Drug Development for Rare Diseases), the targets of drugs in clinical trials are mapped to the KEGG PATHWAY to be grasped on molecular networks. In this study, to understand the relationship between drug targets and disease genes, we mapped them to the KEGG NETWORK (networks) defined as functionally meaningful segments of pathways. We found that disease genes tended to be included in networks characteristic for each disease group, whereas drug targets were mapped to networks common to many disease groups. The number of drugs targeting the networks containing disease genes was small in every disease group. However, because several studies have recently addressed that the drugs targeting proteins with genetic evidence of disease association are more likely to be approved, we confirmed the results using the KEGG NETWORK and integrating the risk genes obtained from the latest GWAS data. The results were clearer and more detailed than those of previous studies, which suggests a direction for future drug development.

## Graphical Abstract

**Graphical Abstract:**
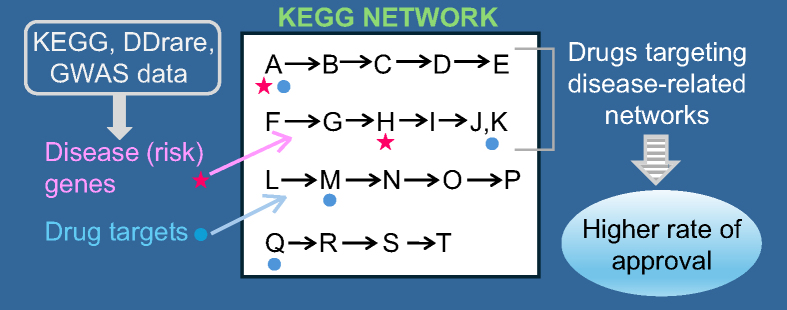

## Introduction

Rare diseases, also termed rare and intractable diseases (RIDs) in Japan, are characterized by a small patient population and a small potential market for drugs. Their pathophysiological mechanisms therefore tend to remain unknown, and the development of drugs against RIDs is not an area of active research. This has resulted in a lack of specific drugs for the treatment of patients with RIDs. Despite many clinical trials involving RIDs that affect a relatively large population, the success rate remains low. We developed the Database of Drug Development for Rare Diseases (DDrare) (https://ddrare.nibiohn.go.jp/), which provides information on drugs tested in clinical trials for RIDs (the intractable diseases designated by the Ministry of Health, Labour and Welfare of Japan). The objective was to understand the trends in drug development to determine the direction to follow, as well as to share the information with the scientific community.

**Figure 1:**
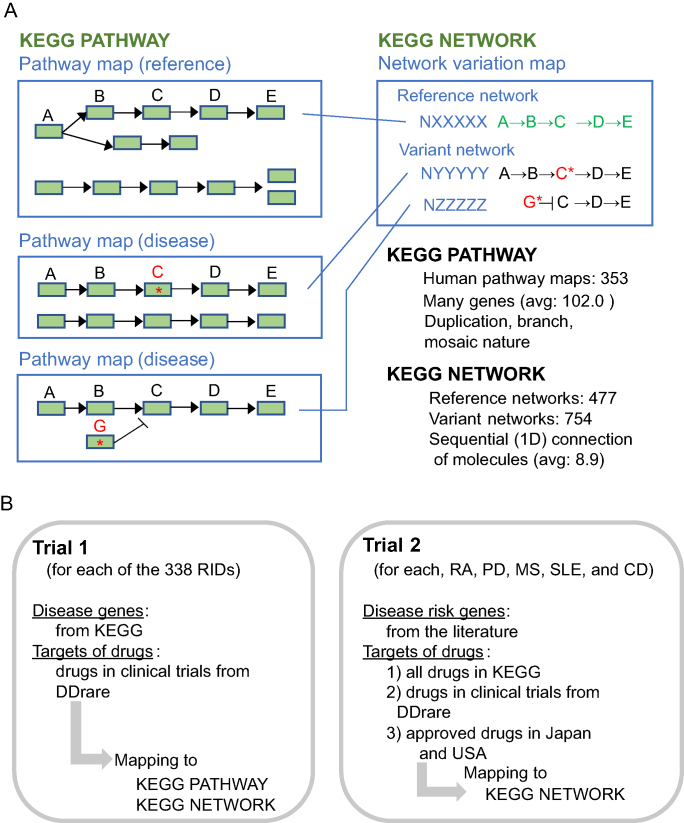
The KEGG PATHWAY, KEGG NETWORK and procedures used to map disease genes and drug targets. (**A**) Relationship between the KEGG PATHWAY and the KEGG NETWORK. The KEGG PATHWAY database includes reference (non-disease) pathway maps and disease pathway maps. In disease pathway maps, genetic alterations are marked in red. The KEGG NETWORK database is a collection of network elements. The two main types of network elements, ‘reference networks’ and ‘disease-related variant (perturbed) networks’, are linked to reference pathway maps and disease pathway maps, respectively. In network variation maps, variant network elements are aligned with reference network elements. (**B**) The procedures used for mapping in this study are shown. Trial 1 was conducted to examine the influence of the large mosaic and overlapping nature of pathway maps on the results. The results were further analyzed to investigate the trends in drug development in each disease group. In trial 2, for each of the diseases, malignant rheumatoid arthritis (RA), Parkinson disease (PD), multiple sclerosis (MS), systemic lupus erythematosus (SLE), Crohn's disease (CD), and ulcerative colitis (UC), the rate of drugs targeting the networks containing disease genes was examined.

Recently, network pharmacology approaches are increasingly being developed and applied to find new drugs and for drug repositioning. Network pharmacology approaches are based on the notion that a disease is rarely the consequence of an abnormality in a single gene, but rather the result of alterations in a complex intracellular network (1); therefore, it is essential to construct human disease-related networks, which address interactions between gene products and cellular metabolites involved in the disease (2,3). One method is to infer context-specific networks directly from disease-specific data. Another popular strategy is *de novo* network enrichment, in which omics data such as gene expression or single-nucleotide variants are projected onto a network for extracting disease modules. In DDrare, we linked the targets of drugs in clinical trials for RIDs to the Kyoto Encyclopedia of Genes and Genomes (KEGG) pathway database (pathway maps) (https://www.kegg.jp/kegg/) (Figure 1A) to comprehend these drugs in molecular networks. However, because of the large mosaic and overlapping nature of pathway maps, it is not clear which one or which combination of multiple biological processes might be perturbed if a pathway map shows a high enrichment score for specific diseases (4,5). In *de novo* network enrichment methods, omics data are also projected onto protein-protein interaction (PPI) networks (6,7). Although the number of identified human PPIs is expanding rapidly, there are certain limitations such as the lack of context information (e.g. tissue and disease annotations of PPIs), and difficulties in extracting meaningful conclusions from PPI networks (8).

The KEGG NETWORK is a database of network elements, simply called networks, defined as functionally meaningful segments of signaling and other pathways (9,10). This database originally aimed to collect information about disease-related molecular networks that are altered not only by gene variants, but also by viruses and other pathogens, environmental factors, and drugs. Two main types of network elements are reference networks and disease-related variant (perturbed) networks (Figure 1A). Generally, each reference network is contained in one or more reference (non-disease) pathway maps and linked to them, whereas variant networks are limited to disease pathway maps. These are aligned in network variation maps. Unlike the KEGG pathway maps, KEGG networks consist of a small number of molecules connected one-dimensionally.

We hypothesized that these networks could be used for pathway analysis to understand the relationship between drug targets and genes related to RIDs. First, we mapped the disease genes and drug targets in pathway maps as well as networks, and confirmed that mapping to networks would provide more accurate and detailed information about the association between disease genes and drug targets. Then, we performed a detailed analysis of the results of network mapping to determine which networks the disease genes and drug targets belonged to and whether these networks overlapped to identify the trends in drug development for RIDs. A drug target that was on a network together with a disease gene was identified as a drug that targets the specific ‘disease-related network’. Although drugs that target proteins with direct genetic evidence of disease association and their molecular partners are more likely to be approved (11–13), the effects of genetic evidence are likely to be underestimated because the range of the disease-relevant dysfunction in a molecular network tended to be limitedly defined in former studies. We applied the mapping of disease genes and drug targets of six RIDs to the KEGG NETWORK and examined the degree to which the proportion of drugs targeting disease-related networks increases across the drug development pipelines, namely, all drugs, drugs in clinical trials, and approved drugs. Recent publications (14,12) have highlighted the value of genetic information from genome-wide association studies (GWAS) as well as Mendelian inheritance for the identification and prioritization of potential targets (15). Therefore, we added the latest GWAS data to disease gene data in the mapping of them to the KEGG NETWORK for above-mentioned comparison. The results suggest a future course in drug development.

## Materials and methods

### Data source

Information on pathway maps, networks, genes, disease genes, drugs, and drug targets was collected from KEGG version 106.0 in April 2023 using the KEGG API (Application Programming Interface). The information included the lists of materials as well as the associations between them such as pathway maps and genes, networks and genes, and drugs and their targets. Data on RIDs and clinical trial data were obtained from DDrare version March 2022. RID genes were collected from KEGG DISEASE entry pages, which are based on Online Mendelian Inheritance in Man (OMIM) (16), whereas risk genes for the six RIDs included were extracted from the literature related to the GWAS catalog (17). Data for each of the six GWAS are available to download from the websites of the corresponding studies:

Malignant rheumatoid arthritis (RA): (18)

Parkinson disease (PD): (19,20)

Multiple sclerosis (MS): (21)

Systemic lupus erythematosus (SLE): (22)

Crohn disease (CD), ulcerative colitis (UC): (23)

These six RIDs were selected for accurate calculation of the ratio of drugs targeting networks. At first, we chose 9 RIDs with the most numerous drugs in clinical trials. Next, we excluded a RID with least approved drugs (i.e. amyotrophic lateral sclerosis) and two RIDs with least GWAS data (i.e. cystic fibrosis and scleroderma) among the 9 RIDs. All 338 RIDs were classified into 15 categories according to the guidelines of the Japan Intractable Disease Information Center in April 2022 (https://www.nanbyou.or.jp/).

### Calculation of the number of pathways/networks containing disease genes and/or drug targets

The number of pathway maps/networks containing disease genes and/or drug targets were calculated as follows:

The datasets of human pathway maps, networks, and drugs were defined as P = {P_1_, P_2_, P_3_,…., P_353_}, N = {N_1_, N_2_, N_3_,…., N_1260_}, Dr = {Dr_1_, Dr_2_, Dr_3_, …, Dr_12273_}, respectively. Each element of the sets is also a set of genes contained in the pathway map, network, or drug targets: P*_i_* = {g_1_, g_2_, g_3_, ….}, N*_i_* = {g_1_, g_2_, g_3_, ….}, Dr*_i_* = {g_1_, g_2_, g_3_, ….}. The datasets of disease genes and drugs for a disease were defined as Dg*_j_* = {g_1_, g_2_, g_3_,….g}, Dd*_j_* = {d_1_, d_2_, d_3_,….}, respectively, where *j* is the disease number (i.e. an ID of rare and intractable diseases). The set of targets of drug d_i_ in Dd*_j_* was defined as T*_ij_* = {t_1_, t_2_, t_3_, ….}.

The *SPG_i_* score was defined as the summation of the number of overlapping genes between disease *j* and pathway *i* in all diseases as shown in Eq. (1):


(1) \begin{eqnarray*}SP{{G}_i}\ = \ \mathop \sum \limits_{j\ = \ 1}^n |{{{\mathrm{P}}}_i} \cap {\mathrm{D}}{{{\mathrm{g}}}_j}|\end{eqnarray*}

*n* is the number of all diseases (i.e. 338).

*PG* was defined as the number of pathway maps containing disease genes:


(2) \begin{eqnarray*}\begin{array}{@{}l@{}} \ PG = \mathop \sum \limits_{i = 1}^n {{E}_i}\\ \left( \begin{array}{@{}rcl@{}} {{E}_i} &=& 1{\mathrm{ }}\left( {SP{{G}_i}_{} >{\mathrm{ }}0} \right)\\ &=& 0{\mathrm{ }}\left( {SP{{G}_i}_{} = {\mathrm{ }}0} \right) \end{array} \right) \end{array}\end{eqnarray*}

where *n* is the number of all human pathway maps (i.e. 353).

The *SPT_i_* score was defined as the summation of the number of overlapping genes between the drug targets for a disease and a pathway in all diseases as shown in Eq. (3):


(3) \begin{eqnarray*}SP{{T}_i}\ = \ \mathop \sum \limits_{j\ = \ 1}^n |{{{\mathrm{P}}}_i} \cap {{{\mathrm{T}}}_j}|\end{eqnarray*}

where ${{{\mathrm{T}}}_j}$ is the union of the target genes of drugs for disease j: ${{{\mathrm{T}}}_j} = {{{\mathrm{T}}}_{1j}}\ \cup{{{\mathrm{T}}}_{2j}}\cup{{{\mathrm{T}}}_{3j}} \ldots \ldots \ldots \ldots$

*n* is the number of all diseases (i.e. 338).

*PT* was defined as the number of pathway maps containing drug targets:


(4) \begin{eqnarray*}\begin{array}{@{}l@{}} \ PT = \mathop \sum \limits_{i = 1}^n {{E}_i}\\ \left( \begin{array}{@{}rcl@{}} {{E}_i} &=& 1{\mathrm{ }}\left( {SP{{T}_i}_{} >{\mathrm{ }}0} \right)\\ &=& 0{\mathrm{ }}\left( {SP{{T}_i}_{} = {\mathrm{ }}0} \right) \end{array} \right) \end{array}\end{eqnarray*}

where *n* is the number of all human pathway maps (i.e. 353).

*PGT* was defined as the number of pathway maps containing both disease genes and drug targets:


(5) \begin{eqnarray*}\begin{array}{@{}l@{}} PGT = \mathop \sum \limits_{i = 1}^n {{E}_i}\ \\ \,\,\, \left( \begin{array}{@{}rcl@{}} {{E}_i} &=& 1{\mathrm{ }}\left( {SP{{G}_i}_{} >{\mathrm{ }}0\ {\mathrm{ }}and\ SP{{T}_i} > {\mathrm{ }}0} \right)\\ &=& 0{\mathrm{ }}\left( {else} \right) \end{array} \right) \end{array}\end{eqnarray*}

where *n* is the number of all human pathway maps (i.e. 353).

The final score was defined as the number of diseases with overlapping genes between the disease gene and a pathway as well as the overlapping genes between the drug targets of the disease and the pathway as shown in Eq. (6):


(6) \begin{eqnarray*}\begin{array}{@{}l@{}}\ \ \ \, {{S}_{i\ }} = \mathop \sum \limits_{j = 1}^n {{\alpha }_{ij}}\\ \left( \begin{array}{@{}rcl@{}} {{\alpha }_i}_j &=& 1{\mathrm{ }}(|{\rm P}_i\cap {\rm DG}_i| >{\mathrm{ }}0\ and\ |{\rm P}_i\cap {\rm T}_j| > {\mathrm{ }}0)\\ &=& {\mathrm{ }}0{\mathrm{ }}\left( {else} \right) \end{array} \right) \end{array}\end{eqnarray*}

where *n* is the number of all diseases (i.e. 338).

*PGT_s* was defined as the number of pathway maps containing the sets of disease genes and drug targets in the same disease:


(7) \begin{eqnarray*}\begin{array}{@{}l@{}} PGT\_s = \mathop \sum \limits_{i = 1}^n {{E}_i}_{\ }\\ \,\,\quad \left( \begin{array}{@{}rcl@{}} {{E}_i} &=& 1{\mathrm{ }}\left( {{{S}_i}_{} >{\mathrm{ }}0} \right)\\ &=& 0{\mathrm{ }}\left( {{{S}_i} = {\mathrm{ }}0} \right) \end{array} \right) \end{array}\end{eqnarray*}

where *n* is the number of all human pathway maps (i.e. 353).

Similarly, the number of networks containing disease genes (i.e. *NG*), drug targets (i.e. *NT*), both disease genes and drug targets (i.e. *NGT*), and the sets of disease genes and drug targets in the same disease (i.e. *NGT_s*) were calculated. The number of all networks was 1260. The calculation programs were written in python.

### Calculation of the final score of a network containing disease genes and/or drug targets

The final score of a network (*i*) containing disease (*j*) genes was defined as the summation of the number of overlapping genes between disease genes and a network in each disease group as described in Eq. (8):


(8) \begin{eqnarray*}SN{{G}_i} = \mathop \sum \limits_{j = 1}^n |{{{\mathrm{N}}}_i} \cap {\mathrm{D}}{{{\mathrm{g}}}_j}\ |{\mathrm{\ }}\end{eqnarray*}

where *n* is the number of diseases in the disease group.

The final score of a network containing drug targets was defined as the summation of the number of drugs targeting proteins encoded by the genes in a network in each disease group as described in Eq. (9):


(9) \begin{eqnarray*}SN{{D}_{i\ }} = \mathop \sum \limits_{j = 1}^n {{\beta }_{ij}}{\mathrm{\ }}\end{eqnarray*}

where ${{\beta }_{ij}}$ is the number of drugs for disease *j* that have overlapping genes between drug targets and network *i*; n is the number of diseases in the disease group:


(10) \begin{eqnarray*}\ {{\beta }_{ij}} = \ |\left\{ {{\mathrm{D}}{{{\mathrm{r}}}_k}:|({{{\mathrm{N}}}_i} \cap {{{\mathrm{T}}}_j}) \cap {\mathrm{D}}{{{\mathrm{r}}}_k}| >0} \right\} \cap {\mathrm{D}}{{{\mathrm{d}}}_j}|\end{eqnarray*}

Then, the networks were ranked according to the scores (*SNG_i_* or *SND_i_*).

### Calculation of the number of drugs targeting the networks containing disease risk genes

To calculate the number of drugs targeting the networks containing disease risk genes for each of the six RIDs, we defined the datasets of networks containing both disease genes and drug targets of a disease as described in Eq. (11):


(11) \begin{eqnarray*}{\mathrm{N}}{{{\mathrm{C}}}_j} = \left\{ {{{{\mathrm{N}}}_i}:|{{{\mathrm{N}}}_i} \cap {\mathrm{D}}{{{\mathrm{g}}}_j}| >0\ {\mathrm{and}}\ |{{{\mathrm{N}}}_i} \cap {{{\mathrm{T}}}_j}| > 0\ } \right\}\ \end{eqnarray*}

where *j* is the disease number (i.e. 46, 6, 13, 49, 96, and 97).

For each element of NC*_j_*, namely N*_i_*, the dataset of drugs targeting the network was defined as described in Eq. (12):


(12) \begin{eqnarray*}{\mathrm{DN}}{{{\mathrm{C}}}_{ij}} = \{ {\mathrm{D}}{{{\mathrm{r}}}_k} :|({{{\mathrm{N}}}_i} \cap {{{\mathrm{T}}}_j}) \cap {\mathrm{D}}{{{\mathrm{r}}}_k}| >0\} \cap {\mathrm{D}}{{{\mathrm{d}}}_j}\end{eqnarray*}

The number of drugs targeting the networks containing disease risk genes for each of the six RIDs was defined as described in Eq. (13):


(13) \begin{eqnarray*}{\mathrm{DNG}}{{{\mathrm{T}}}_j}\_s\ = \ \left| {{\mathrm{DN}}{{{\mathrm{C}}}_{1j}}\cup{\mathrm{DN}}{{{\mathrm{C}}}_{2j}}\cup{\mathrm{DN}}{{{\mathrm{C}}}_{3j}} \ldots \ldots \ldots } \right|\end{eqnarray*}

Among Dr*_k_* in Eq. (12), the drugs whose targets overlapped with the risk genes were regarded as the drugs targeting the disease genes themselves.

The dataset of drugs targeting any network in the KEGG NETWORK was defined as described in Eq. (14):


(14) \begin{eqnarray*}{\mathrm{DN}}{{{\mathrm{A}}}_j} = \{ {\mathrm{D}}{{{\mathrm{r}}}_k} :\left| {{\mathrm{NA}} \cap {\mathrm{D}}{{{\mathrm{r}}}_k}} \right| >0\} \cap {\mathrm{D}}{{{\mathrm{d}}}_j}\end{eqnarray*}

where NA is the union of the genes contained in all networks: ${\mathrm{NA\ }} = {{{\mathrm{N}}}_1}\ \cup{{{\mathrm{N}}}_2}\cup{{{\mathrm{N}}}_3} \ldots \ldots \ldots {{{\mathrm{N}}}_{1260}}$

### Statistical analysis

Statistical analysis was performed using Excel statistical analysis software (BellCurve for Excel). The relationship between two categorical variables was analyzed using the Chi-square test. Differences between three categorical variables were analyzed using three-way analysis of variance (ANOVA). When the ANOVA was significant, follow-up post hoc comparisons (Tukey's HSD) were performed. * *P* < 0.05, ** *P* < 0.01, *** *P* < 0.001, **** *P* < 0.0001.

## Results

### Comparison between the KEGG PATHWAY and the KEGG NETWORK

**Figure 2:**
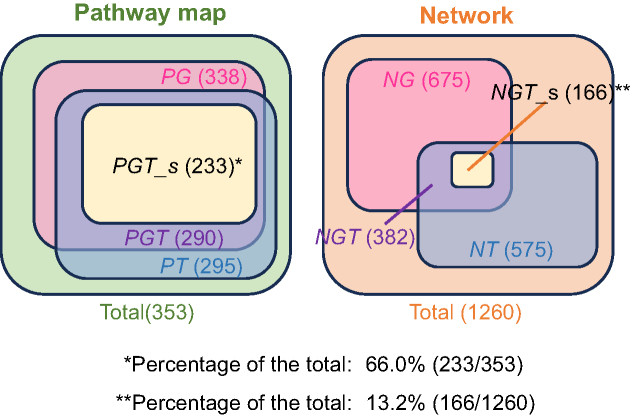
Venn diagram showing the numbers of pathway maps (**A**) and networks (**B**). The numbers of pathway maps containing disease genes (*PG*), drug targets (*PT*), both of them (*PGT*), or the sets of disease genes and drug targets in the same disease (*PGT_s*) are shown in parenthesis. The numbers of networks containing disease genes (*NG*), drug targets (*NT*), both of them (*NGT*), or the sets of disease genes and drug targets in the same disease (*NGT_s*) are also the same.

To examine the influence of the large mosaic and overlapping nature of pathway maps on the results of gene mapping, we first mapped disease genes and the targets of drugs in clinical trials to pathway maps and networks and compared them (Figure 1B, left). The results are shown in Figure 2. The number of pathway maps containing disease genes, drug targets, or both did not differ, whereas there was a significant difference in the number of networks to which these gene sets were mapped. The chi-square test of independence showed a statistically significant relationship (****P < 0.0001) between the type of molecular network (i.e. pathway map or network) and the number of elements containing disease genes and/or drug targets (Supplementary Table S1). It is possible that the large and overlapping nature of pathway maps was reflected in the results. Because there are many parallel pathways and branches of a pathway in a pathway map (Figure 1A), the coexistence of disease genes and drug targets in a pathway map does not necessarily suggest a direct relationship between them in a molecular network. Moreover, even if disease genes and drug targets are in a molecular network, the same network could be contained in many pathway maps. In such a case, the biological implications of the relationship between disease genes and drug targets may be obscured by the various names of pathway maps containing them. On the other hand, when the disease genes and the targets of drugs for the disease are mapped to the same network, the genes and drug targets are likely to have a mutual relationship in the molecular network, and the limited significance of the network may be reflected in its name. Therefore, we confirmed that the mapping of disease genes and drug targets to networks provides a more accurate and detailed understanding of the relationship between them than mapping to pathway maps.

### KEGG networks containing disease genes and/or drug targets

**Figure 3:**
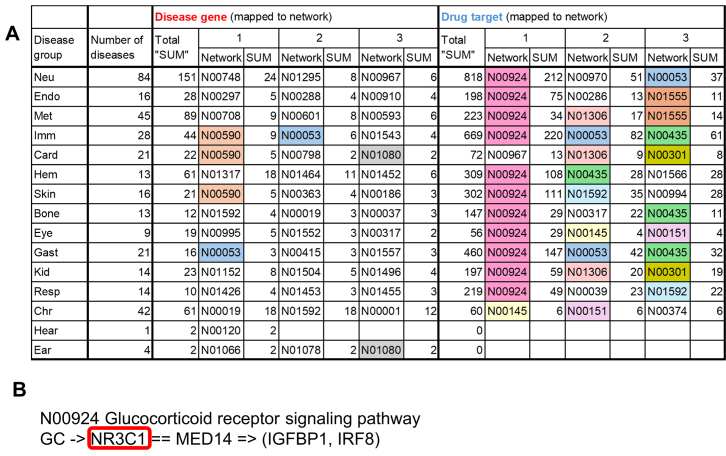
Reference networks mapped with many disease genes or drug targets in each disease group. (**A**) Table showing networks ranked on the basis of the scores obtained by summing the number of overlapping genes between disease genes and the networks or those of drugs targeting each network in each disease group. ‘Total SUM’ is calculated by adding the number of drugs for each disease that target any network in KEGG NETWORK (see Eq. (14) in Materials and methods). Overlapping networks in several disease groups are shown in different colors according to the networks (i.e. N00590: antigen processing and presentation by MHC class II molecules; N00053: cytokine–Jak–STAT signaling pathway; N01080: ITGA/B-TALIN/VINCULIN signaling pathway; N00924: glucocorticoid receptor signaling pathway; N01555: hormone-like-cytokine to Jak–STAT signaling pathway; N01306: AngII–AT1R–NOX2 signaling pathway; N00435: TLR1/2/4-NFKB signaling pathway; N00301: angiotensin–aldosterone signaling pathway; N01592: GF–RTK–RAS–ERK signaling pathway; N00145: extrinsic apoptotic pathway; N00151: TNF–NFκB signaling pathway). Neu, neuromuscular diseases; Endo, endocrine diseases; Met, metabolic diseases; Imm, immune diseases; Card, cardiovascular diseases; Hem, hematologic diseases; Skin, skin and connective tissue diseases; Bone, bone and joint diseases; Eye, eye diseases; Gast, gastrointestinal diseases; Kid, kidney and urinary diseases; Resp, respiratory diseases; Chr, chromosomal and gene abnormalities; Hear, hearing and balance disorders; Ear, ear, nose, and throat diseases. (**B**) The top network mapped with many drug targets. Red frame indicates the drug target.

To investigate the trends in drug development for RIDs, we analyzed the results obtained in the former section in detail. The top three networks ranked according to the score indicating the total number of mapped genes in each disease group are shown in Figure 3. In terms of the networks containing disease genes, there was little overlap among disease groups. The classification of networks according to the pathway maps containing them showed that the networks containing disease genes tended to be characteristic of the disease group: the top three networks of endocrine diseases, metabolic diseases, and immune diseases were contained in the pathway maps of ‘5.2 Endocrine system’, ‘1. Metabolism’ and ‘5.1 Immune system’ in the KEGG PATHWAY, respectively. On the other hand, the networks containing drug targets were common to many disease groups: the top network, N00924 ‘Glucocorticoid receptor signaling pathway’, in which all targeted proteins were encoded by NR3C1, was included in 11 disease groups. In addition, N00053 ‘Cytokine-Jak-STAT signaling pathway’, N01306 ‘AngII-AT1R-NOX2 signaling pathway’ and N00435 ‘TLR1/2/4-NFKB signaling pathway’ were also highly ranked in several disease groups. According to the Anatomical Therapeutic Chemical (ATC) classification (Supplementary Table S2), the drugs targeting NR3C1 in N00924 were corticosteroids, which are anti-inflammatory agents. These were developed for 88 of the 338 RIDs. Likewise, the drugs targeting N00053, N01306 or N00435 were immunosuppressants, and were developed for 117 of the 338 RIDs. Although the diseases for which these drugs were developed were included in various disease groups, many of them were autoimmune diseases (the former: 35 out of 88 diseases, the latter: 32 out of 117 diseases. According to ‘Global Autoimmune Institute’: https://www.autoimmuneinstitute.org/).

**Figure 4:**
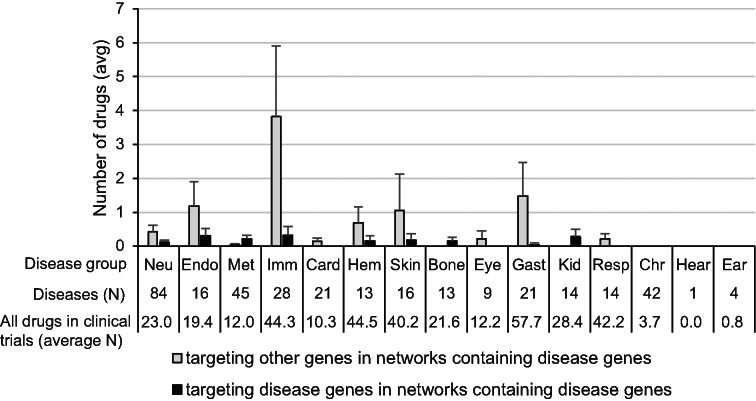
Average number of drugs targeting the networks containing the disease genes in each disease group. The data are presented as mean ± SE. The number of diseases included and the average number of drugs in clinical trials in each group are also shown under the bars. The three-letter codes of disease groups are same as those in Figure 3.

**Figure 5:**
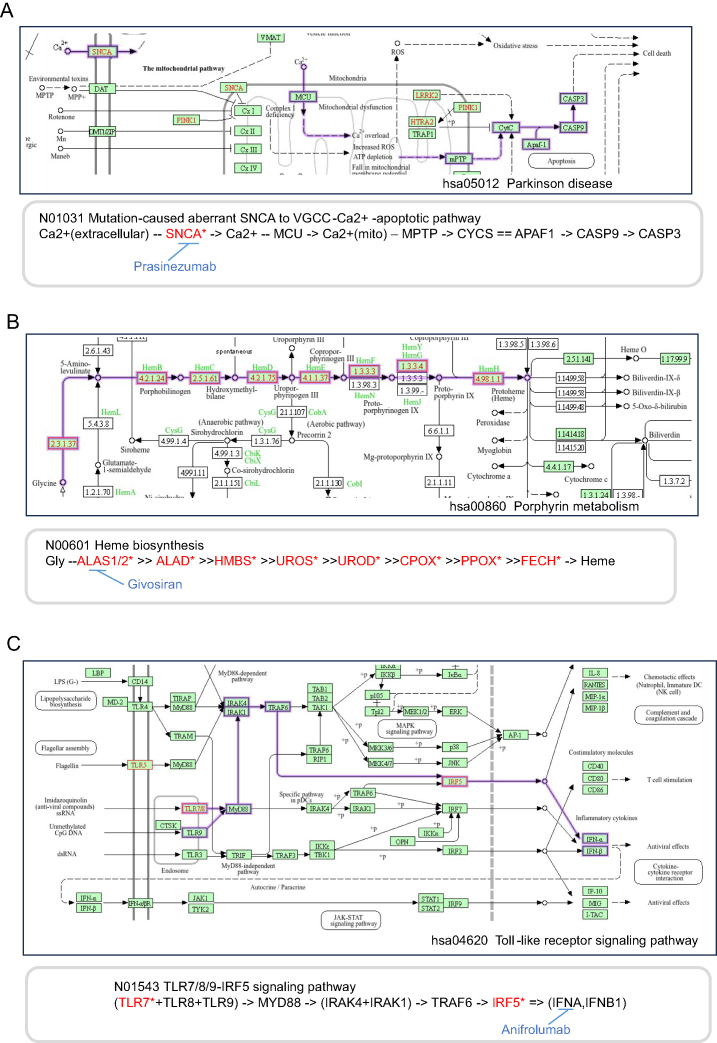
Examples of drugs targeting the networks containing disease genes. (**A**: Parkinson disease. **B**: Porphyria. **C**: Systemic lupus erythematosus). The networks are linked to pathway maps. The network of A is a variant network and those of B and C are reference networks. Disease genes are marked in red on both the pathway maps and the networks. The drugs targeting the component of the networks are displayed in blue. Edges represent interactions/reactions: -- (node) ->, transport through the node; = = , complex formation; ->, activation; --, substrate-enzyme relation; >>, enzyme-enzyme relation; = >, expression

The number of drugs targeting the proteins encoded by disease genes themselves or other components of the networks containing disease genes was too small in every disease group (Figure 4 and Supplementary Figure S1). Examples of drugs with targets mapped to pathway maps and networks are shown in Figure 5.

(Parkinson disease)

Fibrillar alpha-synuclein is a component of the Lewy body, the characteristic neuronal inclusion of the PD brain, and mutations in alpha-synuclein cause autosomal-dominant hereditary PD. Studies suggest that mutant alpha-synuclein generates ion pores that induce Ca^2+^ influx into neurons and therefore plays an important role in cell degeneration (24–26). Prasinezumab is a monoclonal antibody that specifically targets aggregated alpha-synuclein and is in clinical development.

(Porphyria)

The porphyrias are a group of diseases, each resulting from a defect in an enzyme of the heme synthesis pathway. These enzymatic defects cause the accumulation of heme precursors or porphyrins that are responsible for the distinct clinical features of each type of porphyria (27). Givosiran is a small interfering RNA (siRNA) that targets ALAS1 in hepatocytes, thereby preventing the accumulation of delta-aminolevulinic acid (ALA) and porphobilinogen (PBG) (28,29).

(SLE)

Many of the genetic variants associated with SLE encode proteins involved in the activation or regulation of the innate immune response, and some of these (e.g. TLR7, IRF5) mediate the production of type I interferon (IFN-I) (30,31). Therefore, to inhibit the IFN-I pathway, monoclonal antibodies (e.g. rontalizumab, sifalimumab) that block IFN-α have been developed.

In this way, drugs that target not only the proteins encoded by the disease genes and the proteins interacting with them directly, but also the proteins upstream of those encoded by disease genes in metabolic pathways or the downstream proteins whose expression is regulated by the disease genes were extracted by mapping them to networks.

### Rate of drugs targeting networks containing disease genes

**Figure 6:**
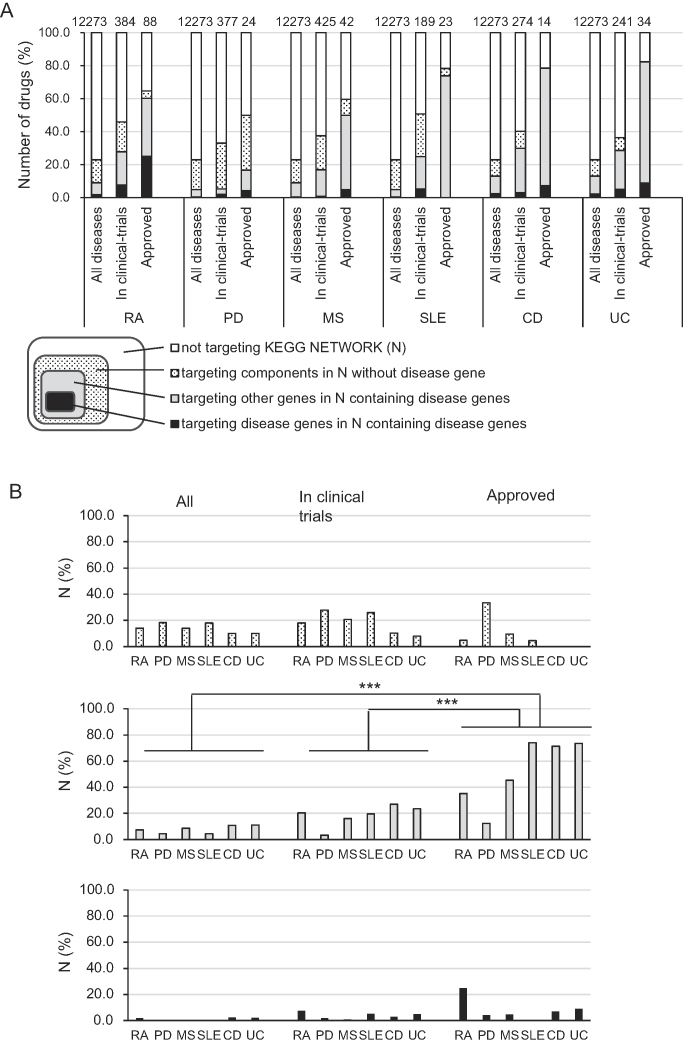
Rate of drugs targeting the disease-related networks in each of the six RIDs. (**A**) Changes in the proportion of drugs for each of the six RIDs targeting or not-targeting the disease-related networks across the drug development pipelines, namely, ‘all drugs’, ‘drugs in clinical trials’ and ‘approved drugs’ are shown in the bar graph. All ‘drugs in clinical trials’ are included in ‘all drugs’, and ‘approved drugs’ are generally included in ‘drugs in clinical trials.’ Not all ‘approved drugs’ are included in ‘drugs in clinical trials’ because there are clinical trial registries that are not registered in DDrare. The upper number of each bar is the total number of drugs. The lower diagram represents the association between the drug groups. (**B**) The divided bar graphs of A show the results of three way ANOVA with the factors DISEASE (RA, PD, MS, SLE, CD, UC), DRUG-DEVELOPMENT PHASE (all, in clinical trials, approved), and TARGET (targeting components in networks without disease genes, targeting other genes in networks containing disease genes, targeting disease genes in networks containing disease genes) and the percentage of drugs as the dependent variable. There are significant interaction effects for DRUG-DEVELOPMENT PHASE × TARGET (*F* = 11.8, ***P < 0.001). The statistical significance of differences between drug-development phases are shown: ***P < 0.001 by post-hoc Tukey's tests. RA, malignant rheumatoid arthritis; PD, Parkinson disease; MS, multiple sclerosis; SLE, systemic lupus erythematosus; CD, Crohn's disease; UC, ulcerative colitis.

To determine how the proportion of drugs targeting disease-related networks changes in association with drug development pipelines, namely, all drugs, drugs in clinical trials, and approved drugs, we mapped disease genes and drug targets of several RIDs onto the KEGG NETWORK (Figure 1B, right). Diseases affecting a relatively large population are the subject of many clinical trials (Supplementary Figure S2). Because a certain number of drugs are needed for statistical analysis, we selected malignant RA, PD, MS, SLE, CD and UC among 338 RIDs. In this section, we integrated KEGG DISEASE genes mainly extracted from OMIM and risk genes obtained from GWAS as ‘disease genes’. For drugs, ‘all drugs’ indicated all drugs registered in KEGG DRUG and ‘approved drugs’ were defined as a combination of drugs extracted from Japanese drug labels and FDA drug labels (Supplementary Table S3). The results of mapping are shown in Figure 6A. Statistical analysis was performed by three way ANOVA with the factors DISEASE (RA, PD, MS, SLE, CD, UC), DRUG-DEVELOPMENT PHASE (all, in clinical trials, approved), and TARGET (targeting components in networks without disease genes, targeting other genes in networks containing disease genes, targeting disease genes in networks containing disease genes), and the percentage of drugs was used as the dependent variable. The results of ANOVA were significant for the factors DRUG-DEVELOPMENT PHASE (*F* = 10.8, ***P < 0.001) and TARGET (*F* = 21.1, ***P < 0.001). Significant interaction effects were observed for DISEASE × TARGET (*F* = 2.9, **P = 0.002) and DRUG-DEVELOPMENT PHASE × TARGET (*F* = 11.8, ***P < 0.001). Post-hoc Tukey's tests indicated that for the percentage of drugs targeting genes other than disease genes in the networks containing disease genes, the number of approved drugs was significantly higher than that of all drugs (***P < 0.001) or drugs in clinical trials (***P < 0.001) (Figure 6B). Similar results were obtained after compiling the data for drugs targeting disease genes and other genes in networks containing disease genes. This indicates that there were significant main effects and interaction effects for the factors listed above, and post-hoc Tukey's tests indicated that for drugs targeting the genes in the networks containing disease genes, the rate of approved drugs was significantly higher than that of all drugs (***P < 0.001) or drugs in clinical trials (***P < 0.001). When the term ‘disease genes’ was limited to the genes collected from KEGG DISEASE entry pages that are mainly based on OMIM, among drugs targeting the components of networks without KEGG disease genes, the percentage of approved drugs was significantly higher than that of all drugs (Tukey's tests ***P < 0.001) or drugs in clinical trials (Tukey's tests ***P < 0.001) (Supplementary Figure S3). Regarding the percentage of drugs targeting genes other than disease genes in the networks containing disease genes, the rate of approved drugs was significantly higher than that of all drugs as well; however, the tendency was not as clear as the result obtained using the integrated KEGG DISEASE genes with the risk genes obtained from GWAS as ‘disease genes’.

## Discussion

We mapped disease genes from 338 RIDs and the targets of drugs in clinical trials to the KEGG PATHWAY and the KEGG NETWORK and confirmed that mapping to the NETWORK yielded more accurate and detailed information about the relationship between disease genes and drug targets. These results suggest that the KEGG NETWORK is useful for pathway analysis of such a relationship. Castresana-Aguirre *et al.* reported that in the functional analysis of gene sets by pathway annotation, gene sets are often complex and associated with multiple affected pathways (32). In such cases, the association of a gene with a pathway is weakened by the presence of genes associated with other pathways. Therefore, they clustered the query gene set into homogenous components before the analysis of KEGG pathways and found that clustering increases the sensitivity of pathway analysis methods and provides a deeper insight into the biological mechanisms. In the present study, we maintained the queries unchanged and mapped to networks that were, so to speak, divided elements of pathway maps, which proved to be effective. In metabolic pathways, there are KEGG modules that are manually defined as functional units (10). Karp *et al.* indicated that although KEGG modules overcome the problems of pathway maps, they are still incomplete (5). KEGG networks, which were developed after KEGG modules, are similar to KEGG modules because they are defined as functionally meaningful segments and although they are incomplete, they cover the primary signaling pathways as well as metabolic pathways.

The three most highly ranked networks to which many disease genes were mapped tended to differ according to the disease group. The network variation maps in the KEGG NETWORK are computationally drawn diagrams of network variations containing aligned sets of reference network elements and experimentally observed variant (perturbed) network elements. The reference network elements in each network variation map of diseases are the molecular networks that would be perturbed by gene variants, pathogens, and environmental factors involved in the disease (9,10). In the network variation maps, the reference network elements are characteristic of each disease group, such as cancers and neurodegenerative diseases, and are closely related to the pathological features of the disease group. Although most of the RIDs analyzed in this study do not have a network variation map in KEGG, the results show that each disease group among RIDs also has characteristic reference network elements, suggesting that the onset mechanisms differ according to the disease group.

By contrast, the highly ranked networks to which many drug targets were mapped tended to be the same in most disease groups. The network N00924 ‘Glucocorticoid receptor signaling pathway’ had particularly high score and the networks targeted by the drugs categorized to immunosuppressants were also highly ranked. The results suggest that, for diseases associated with inflammation such as autoimmune diseases, there is relatively large number of drugs, many of which are corticosteroids that are necessary for symptomatic relief (33), but are not directly related to disease genes.

Drugs targeting networks containing disease genes, namely, disease-related networks, were also identified, although few of them were in clinical trials. Recent studies (11,14,12) suggest that drugs that target proteins with genetic support are more likely to be therapeutically valid and approved. Nelson (11) reported that the proportion of drug mechanisms with direct genetic support increases significantly across the drug development pipeline, from 2.0% at the preclinical stage to 8.2% among mechanisms for approved drugs. Okada *et al.* found that the pharmacologically active targets of approved RA drugs were not only the protein-products of the biological RA risk genes, but also genes in direct PPI with them. (12). They compared the overlap and relative enrichment of RA risk genes (in addition to genes in direct PPIs with them) with targets of approved RA drugs and with all drug target genes, and calculated the relative fold enrichment as 2.2. Because approved or potential drugs targeting other components of disease-related networks in RIDs are also known (34), we examined six RIDs by mapping drug targets and risk genes to networks. The results showed that the rate of drugs targeting other genes in networks containing disease genes increased more than 4.5 times across the drug development pipeline in 5 of 6 RIDs, which is higher amplification than previous studies, although these cannot simply be compared and there are differences between diseases. This result is consistent with the study by Fang *et al.*, which showed that interacting neighbors rather than GWAS-reported genes are known drug targets, and integrating data on network connectivity increases the informativeness of genetics for target validation (14). The higher rate of change in the proportion of drugs with genetic support across the drug development pipeline in this study might be due to some points: extension of the range of disease-relevant dysfunction from disease genes and genes in direct PPI with them to molecular networks; addition of the relationship between proteins other than direct interactions, namely, transcription factors and their target genes, sequence of enzymes catalyzing a series of chemical reactions in a metabolic pathway, and so on; addition of the latest GWAS data as disease risk genes. However, only the use of networks, which are small one-dimensional segments of signaling and other pathways, provides an understanding of the characteristics of approved drugs. For most of these six RIDs, the rate of patients with a familial history is <10% (35,36), and the odds ratio of each risk gene is generally low. Therefore, patients with the disease (risk) genes might only account for a small proportion of the population analyzed. The fact that drugs with genetic support are more likely to be approved could be because the same molecular networks are perturbed by factors other than genetic alterations in the networks. Network variation maps of diseases in the KEGG NETWORK and the recent systems genetics approach identified important pathways or networks perturbed by multiple omics layers (e.g. gene expression and genetic data) (37,38). In another example, many of the PD risk genes (27/52) obtained from gene expression data alone (39) are contained in the network variation map of PD and map to the same networks as the genes associated with familial PD (Supplementary Figure S4). Moreover, the molecular networks perturbed by environmental factors such as viral proteins may also be common with those altered by disease genes. Evidence supports the involvement of Epstein-Barr virus (EBV) in the etiology of MS and SLE (40,41). Some of the disease (risk) genes of MS and SLE (8/87, 9/72, respectively) are present on the network variation map of EBV and map to the same networks as those perturbed by the proteins encoded by EBV genes (Supplementary Figure S5). Although the network ‘glucocorticoid receptor signaling’ targeted by many drugs does not contain autoimmune disease genes (e.g. MS, SLE and RA), dysregulation of glucocorticoid production at a previous step before the network may play a key role in various autoimmune diseases (42). Based on these findings, the fact that a drug targeting a network with disease genes has been approved suggests the perturbation of the network by specific causes, but not always by the disease genes in the network. Nevertheless, the use of molecular networks containing disease (risk) genes is expected to improve drug development.

Although we selected 6 RIDs with the most numerous ‘in clinical trials’ and ‘approved’ drugs, considering also the number of GWAS data, for accurate calculation of the ratio of drugs targeting networks, the approach in this study can probably be applied to other diseases. In GWAS catalog, at least 50 diseases of 338 RIDs have GWAS data as of July 2024 (Supplementary Table S4). When there are sufficiently many patients to obtain GWAS data in a RID, even if the disease is a ‘rare’ one, the risk genes of the disease can be mapped on KEGG NETWORK. It is assumed that the drugs targeting such networks have high probabilities of approval, as shown in this study. Even though the patients of a disease are too small in number to obtain GWAS data, accumulating multi-omics data and investigating environmental factors regarding the disease may assist in identifying perturbed networks, which will furthermore serve to clarify the disease mechanisms needed for drug development. This would require further expansion of databases such as the KEGG NETWORK, namely, functionally meaningful segments of signaling and other pathways based on experimental evidence. Various diseases other than RIDs in Japan, such as cancers, are known to be caused by molecular perturbations leading to pathological cellular behavior (43), and the findings in this study could be applicable to these diseases. Studies on the association between genetic evidence and drug approval mostly focus on immune diseases. Further study is required to extend the analysis over a wide range of diseases and to make comparisons between them.

## Supplementary Material

ugae009_Supplemental_File

## Data Availability

The scripts and data are available in FigShare at https://doi.org/10.6084/m9.figshare.25904107.
