# Supplementary material for: Trends in drug development for rare and intractable diseases based on the KEGG NETWORK
Source: NAR Mol Med. 2024 Aug 9;1(3):ugae009. doi: 10.1093/narmme/ugae009 (PMC12429956; doi:10.1093/narmme/ugae009)
Supplement: ugae009_Supplemental_File [file ugae009_Supplemental_File.pdf]

Supplementary Information

Trends in drug development for rare and intractable diseases based on the KEGG NETWORK

Mao Tanabe, Makoto Hirata & Ryuichi Sakate

CONTENTS	Page No
Table S1: Contingency table for disease gene/target-inclusion data of pathway maps and networks.	3
Table S2: Anatomical Therapeutic Chemical (ATC) Classification (KEGG BRITE br08303) of drugs targeting the components of each of the top three networks in each disease group	4-7
Figure S1: The number of drugs targeting the components of networks	8
Figure S2: A dispersion chart of the number of Med expenses recipients and that of drugs in clinical-trial data.	9
Table S3: The number of disease genes and drug targets of six RIDs.	10
Figure S3: The drugs targeting or not-targeting KEGG NETWORK with disease genes limited to KEGG DISEASE genes in each of 6 RIDs.	11-12
Figure S4: Risk genes of SLE and MS mapped to the network variation map "(nt06165) Epstein-Barr virus"	13-14
Figure S5: PD expression data mapped to the network variation map "(nt06463) Parkinson disease".	15-16
Table S4: The number of patients given "medical care recipient certificate" (FY2021) in Japan (P), drugs in clinical-trial data (D), and GWAS catalog "associations" (July 2024) (G) of 338 RIDs	17-27

Supplementary Table S1: Contingency table for disease gene/target-inclusion data of pathway maps and networks.

	Containing the sets of disease genes and drug targets in the same disease	Containing both disease genes and drug targets	Containing disease genes	Containing drug targets	Total	SUM
Pathway map	233	290	338	295	353	1509
Network	166	382	675	575	1260	3058
SUM	399	672	1013	870	1613	4567

Supplementary Table S2: Anatomical Therapeutic Chemical (ATC) Classification (KEGG BRITE br08303) of drugs targeting the components of each of the top three networks in each disease group.

Disease group	Neu		
Rank (on the basis of the score SND_i given in Eq. 9 in text)	1	2	3
KEGG NETWORK	N00924	N00970	N00053
H SYSTEMIC HORMONAL PREPARATIONS, EXCL. SEX HORMONES AND INSULINS			
H01 PITUITARY AND HYPOTHALAMIC HORMONES AND ANALOGUES	0	0	0
H02 CORTICOSTEROIDS FOR SYSTEMIC USE	34	0	0
H03 THYROID THERAPY	0	0	0
H04 PANCREATIC HORMONES	0	0	0
H05 CALCIUM HOMEOSTASIS	0	0	0
L ANTINEOPLASTIC AND IMMUNOMODULATING AGENTS			
L01 ANTINEOPLASTIC AGENTS	0	0	0
L02 ENDOCRINE THERAPY	0	0	0
L03 IMMUNOSTIMULANTS	0	0	6
L04 IMMUNOSUPPRESSANTS	0	0	8

	Endo			Met			Imm		
	1	2	3	1	2	3	1	2	3
	N00924	N00286	N01555	N00924	N01306	N01555	N00924	N00053	N00435
H01	0	0	5	0	0	3	0	0	0
H02	33	0	0	23	0	0	37	0	0
H03	0	0	0	0	0	0	0	0	0
H04	0	0	0	0	0	0	0	0	0
H05	0	0	0	0	0	0	0	0	0
L01	0	0	0	0	0	0	0	0	0
L02	0	0	0	0	0	0	0	0	0
L03	0	0	0	0	0	1	0	3	0
L04	0	0	0	0	3	1	0	16	10

Supplementary Table S2: (Continued)

Disease group	Card		
Rank (on the basis of the score SND_i given in Eq. 9 in text)	1	2	3
KEGG NETWORK	N00967	N01306	N00301
H SYSTEMIC HORMONAL PREPARATIONS, EXCL. SEX HORMONES AND INSULINS			
H01 PITUITARY AND HYPOTHALAMIC HORMONES AND ANALOGUES	0	0	0
H02 CORTICOSTEROIDS FOR SYSTEMIC USE	0	0	0
H03 THYROID THERAPY	0	0	0
H04 PANCREATIC HORMONES	0	0	0
H05 CALCIUM HOMEOSTASIS	0	0	0
L ANTINEOPLASTIC AND IMMUNOMODULATING AGENTS			
L01 ANTINEOPLASTIC AGENTS	0	0	0
L02 ENDOCRINE THERAPY	0	0	0
L03 IMMUNOSTIMULANTS	0	0	0
L04 IMMUNOSUPPRESSANTS	0	1	0

	Hem			Skin			Bone		
	1	2	3	1	2	3	1	2	3
	N00924	N00435	N01566	N00924	N01592	N00994	N00924	N00317	N00435
H01	0	0	0	0	2	0	0	0	0
H02	24	0	0	30	0	0	29	0	0
H03	0	0	0	0	0	0	0	0	0
H04	0	0	0	0	0	0	0	0	0
H05	0	0	0	0	0	0	0	0	0
L01	0	0	0	0	23	5	0	1	0
L02	0	0	0	0	0	0	0	0	0
L03	0	0	0	0	0	0	0	0	0
L04	0	8	8	0	1	8	0	0	7

Supplementary Table S2: (Continued)

Disease group	Eye		
Rank (on the basis of the score SND_i given in Eq. 9 in text)	1	2	3
KEGG NETWORK	N00924	N00145	N00151
H SYSTEMIC HORMONAL PREPARATIONS, EXCL. SEX HORMONES AND INSULINS			
H01 PITUITARY AND HYPOTHALAMIC HORMONES AND ANALOGUES	0	0	0
H02 CORTICOSTEROIDS FOR SYSTEMIC USE	28	0	0
H03 THYROID THERAPY	0	0	0
H04 PANCREATIC HORMONES	0	0	0
H05 CALCIUM HOMEOSTASIS	0	0	0
L ANTINEOPLASTIC AND IMMUNOMODULATING AGENTS			
L01 ANTINEOPLASTIC AGENTS	0	0	0
L02 ENDOCRINE THERAPY	0	0	0
L03 IMMUNOSTIMULANTS	0	0	0
L04 IMMUNOSUPPRESSANTS	0	3	3

	Gast			Kid			Resp		
	1	2	3	1	2	3	1	2	3
	N00924	N00053	N00435	N00924	N01306	N00301	N00924	N00039	N01592
H01	0	0	0	0	0	0	0	0	0
H02	37	0	0	21	0	0	25	0	0
H03	0	0	0	0	0	0	0	0	0
H04	0	0	0	0	0	0	0	0	0
H05	0	0	0	0	0	0	0	0	0
L01	0	0	0	0	0	0	0	10	10
L02	0	0	0	0	0	0	0	0	0
L03	0	3	0	0	0	0	0	0	0
L04	0	11	8	0	1	0	0	2	0

Supplementary Table S2: (Continued)

Disease group	Chr		
Rank (on the basis of the score SND_i given in Eq. 9 in text)	1	2	3
KEGG NETWORK	N00145	N00151	N00374
H SYSTEMIC HORMONAL PREPARATIONS, EXCL. SEX HORMONES AND INSULINS			
H01 PITUITARY AND HYPOTHALAMIC HORMONES AND ANALOGUES	0	0	0
H02 CORTICOSTEROIDS FOR SYSTEMIC USE	0	0	0
H03 THYROID THERAPY	0	0	0
H04 PANCREATIC HORMONES	0	0	0
H05 CALCIUM HOMEOSTASIS	0	0	0
L ANTINEOPLASTIC AND IMMUNOMODULATING AGENTS			
L01 ANTINEOPLASTIC AGENTS	0	0	0
L02 ENDOCRINE THERAPY	0	0	0
L03 IMMUNOSTIMULANTS	0	0	0
L04 IMMUNOSUPPRESSANTS	5	5	5

The number in each cell indicates that of drugs targeting the components of each of the top three networks ranked on the basis of the score SND_i described in Eq. 9 in the text. The duplication of drugs between diseases was removed. In this table, only two of the first layers of KEGG BRITE “Anatomical Therapeutic Chemical (ATC) classification” (br08303), namely “H SYSTEMIC HORMONAL PREPARATIONS, EXCL. SEX HORMONES AND INSULINS” and “L ANTINEOPLASTIC AND IMMUNOMODULATING AGENTS”, and their second layers are shown as categories. Many of drugs included in “H02 CORTICOSTEROIDS FOR SYSTEMIC USE” overlaps with those in other categories (e.g., “A07 ANTIDIARRHEALS, INTESTINAL ANTIINFLAMMATORY/ANTIINFECTIVE AGENTS”, “C05 VASOPROTECTIVES”, and “D07 CORTICOSTEROIDS, DERMATOLOGICAL PREPARATIONS”).

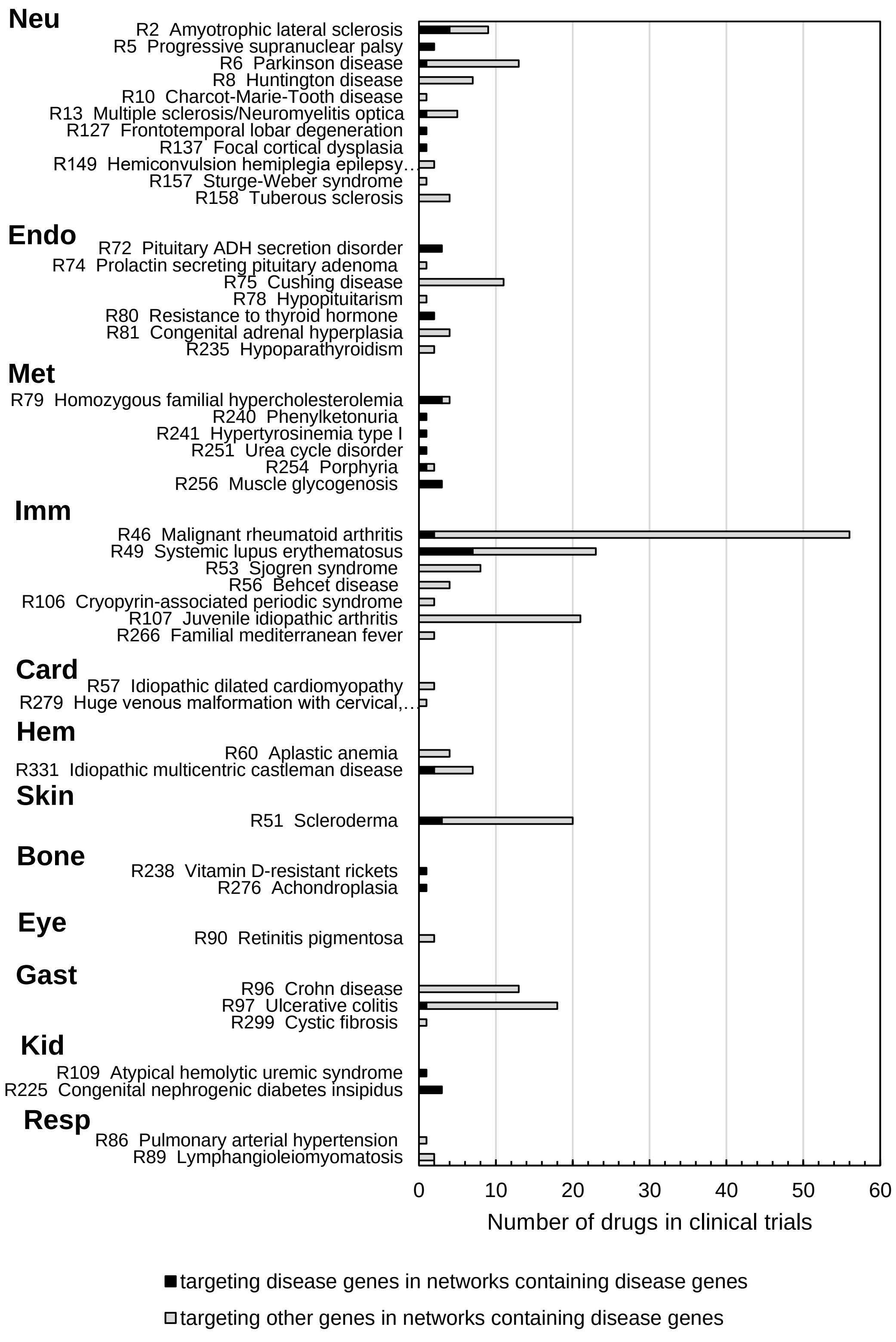

Fig. S1 The number of drugs targeting the networks containing disease genes

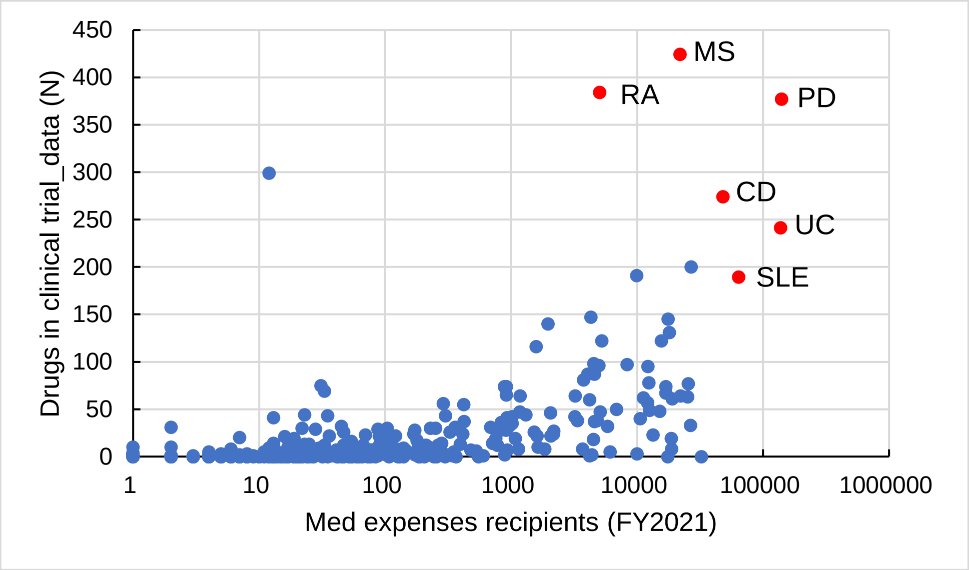

Fig. S2 A dispersion chart of the number of Med expenses recipients in Japan and that of drugs in clinical-trial data. In the graph, all RIDs are plotted. Six red dots indicate the diseases chosen for the analysis of the targets of approved drugs. The graph uses logarithmic scale on the horizontal axis.

Supplementary Table S3: The number of disease genes and drug targets of six RIDs.

Disease				Disease gene			Drug			
ID	KEGG ID	Disease name	Abbr.	KEGG (A)	Others (risk gene)*** (B)	A∪B	DDrare (clinical trial data)	KEGG (approved)		
								Japan (C)	USA (D)	C∪D
46	H00630	Malignant rheumatoid arthritis*	RA	10	152	158	384	69	48	88
6	H00057	Parkinson disease	PD	20	72+38	121	377	24	24	24
13	H01490	Multiple sclerosis	MS	4	84	87	425**	25	34	42
49	H00080	Systemic lupus erythematosus	SLE	23	61	72	189	20	16	23
96	H00286	Crohn disease	CD	7	183	187	274	10	10	14
97	H01466	Ulcerative colitis	UC	4	166	168	241	28	27	34

*Disease name is “rheumatoid arthritis” in KEGG.

**Clinical trial data in DDrare includes the drugs for not only MS but also NMOSD.

***References were mainly chosen from GWAS catalog.

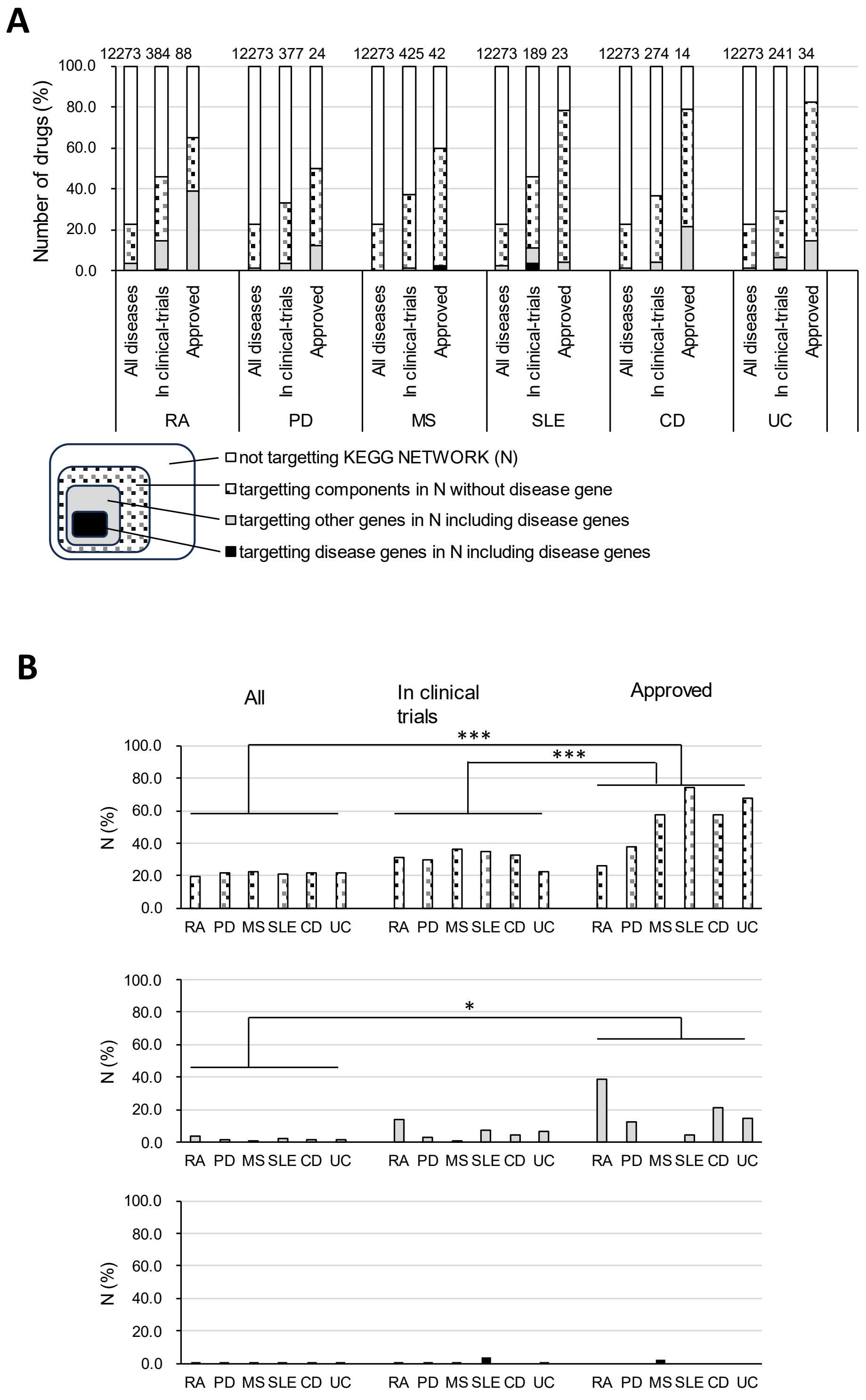
A
12273 384 88
12273 377 24
12273 425 42
12273 189 23
12273 274 14
12273 241 34
Number of drugs (%)
All diseases
In clinical-trials
Approved
RA
PD
MS
SLE
CD
UC
not targetting KEGG NETWORK (N)
targetting components in N without disease gene
targetting other genes in N including disease genes
targetting disease genes in N including disease genes
B
All
In clinical trials
Approved
***
***
*
N (%)
RA PD MS SLE CD UC

Fig. S3 The rate of drugs targeting or not-targeting KEGG NETWORK with disease genes limited to KEGG DISEASE genes in each of 6 RIDs. **A**. The change of the proportion of the drugs for each of 6 RIDs targeting or not-targeting the disease-related networks (containing only KEGG disease genes) across the drug development pipelines, namely, all drugs, drugs in clinical trials, and the approved drugs are shown in the bar graph. The lower diagram represents the inclusion relation between the drug groups. **B**. The divided bar graphs of A to show the results of three way ANOVA with factors DISEASE (RA, PD, MS, SLE, CD, UC), DRUG-DEVELOPMENT PHASE (all, in clinical trials, approved), and TARGET (targeting components in networks without disease gene, targeting other genes in networks containing disease genes, targeting disease genes in networks containing disease genes) with the percentage of drugs as the dependent variable. There are significant main effects for factor DRUG-DEVELOPMENT PHASE (F=16.3, P<0.001) and TARGET (F=93.9, P<0.001). A significant interaction effects are also found for DRUG-DEVELOPMNET PHASE × TARGET (F=5.9, P=0.0026). Post-hoc Tukey's tests indicate significantly higher percentage of approved drugs than that of all drugs (P<0.001) or the drugs in clinical trials (P<0.001) within the drugs targeting the components in networks without KEGG disease gene. Within the drugs targeting the genes other than disease gene in the networks containing KEGG disease genes, the percentage of the approved drugs are higher than that of all drugs (P=0.023).

A

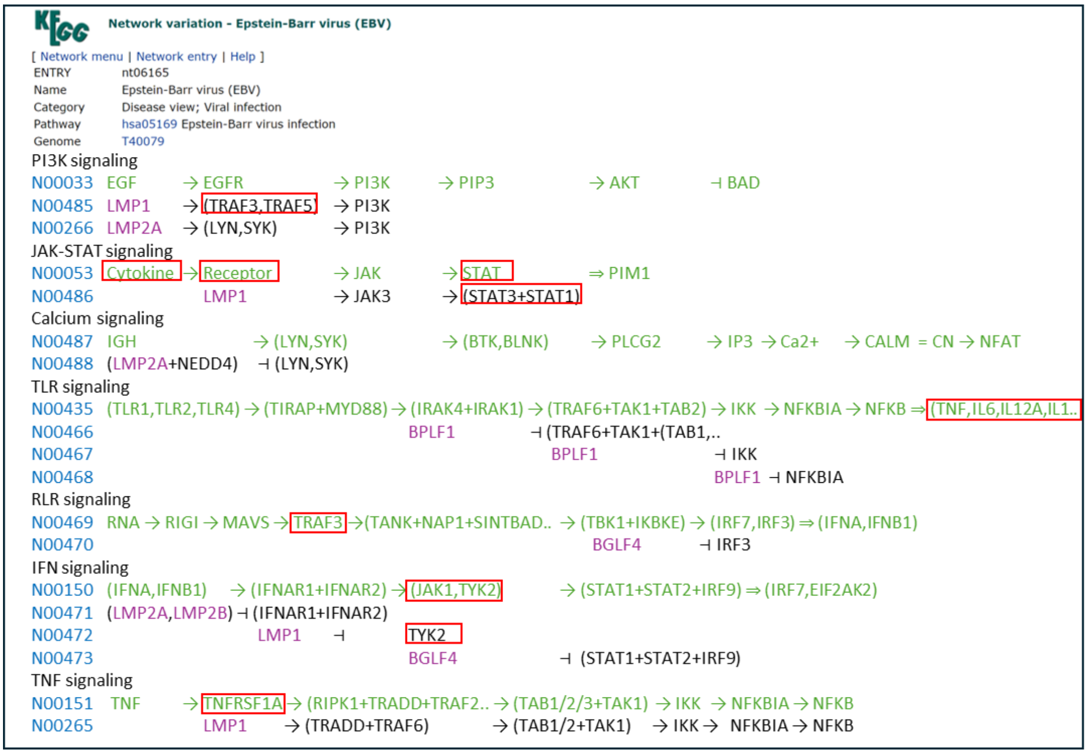

Network variation - Epstein-Barr virus (EBV)
[Network menu | Network entry | Help]
ENTRY nt06165
Name Epstein-Barr virus (EBV)
Category Disease view; Viral infection
Pathway hsa05169 Epstein-Barr virus infection
Genome T40079
PI3K signaling
N00033 EGF → EGFR → PI3K → PIP3 → AKT ⊣ BAD
N00485 LMP1 → (TRAF3,TRAF5) → PI3K
N00266 LMP2A → (LYN,SYK) → PI3K
JAK-STAT signaling
N00053 Cytokine → Receptor → JAK → STAT ⇒ PIM1
N00486 LMP1 → JAK3 → (STAT3+STAT1)
Calcium signaling
N00487 IGH → (LYN,SYK) → (BTK,BLNK) → PLCG2 → IP3 → Ca2+ → CALM = CN → NFAT
N00488 (LMP2A+NEDD4) ⊣ (LYN,SYK)
TLR signaling
N00435 (TLR1,TLR2,TLR4) → (TIRAP+MYD88) → (IRAK4+IRAK1) → (TRAF6+TAK1+TAB2) → IKK → NFKBIA → NFKB ⇒ (TNF,IL6,IL12A,IL1..
N00466 BPLF1 ⊣ (TRAF6+TAK1+(TAB1,..
N00467 BPLF1 ⊣ IKK
N00468 BPLF1 ⊣ NFKBIA
RLR signaling
N00469 RNA → RIGI → MAVS → TRAF3 → (TANK+NAP1+SINTBAD.. → (TBK1+IKBKE) → (IRF7,IRF3) ⇒ (IFNA,IFNB1)
N00470 BGLF4 ⊣ IRF3
IFN signaling
N00150 (IFNA,IFNB1) → (IFNAR1+IFNAR2) → (JAK1,TYK2) → (STAT1+STAT2+IRF9) ⇒ (IRF7,EIF2AK2)
N00471 (LMP2A,LMP2B) ⊣ (IFNAR1+IFNAR2)
N00472 LMP1 ⊣ TYK2
N00473 BGLF4 ⊣ (STAT1+STAT2+IRF9)
TNF signaling
N00151 TNF → TNFRSF1A → (RIPK1+TRADD+TRAF2.. → (TAB1/2/3+TAK1) → IKK → NFKBIA → NFKB
N00265 LMP1 → (TRADD+TRAF6) → (TAB1/2+TAK1) → IKK → NFKBIA → NFKB

B

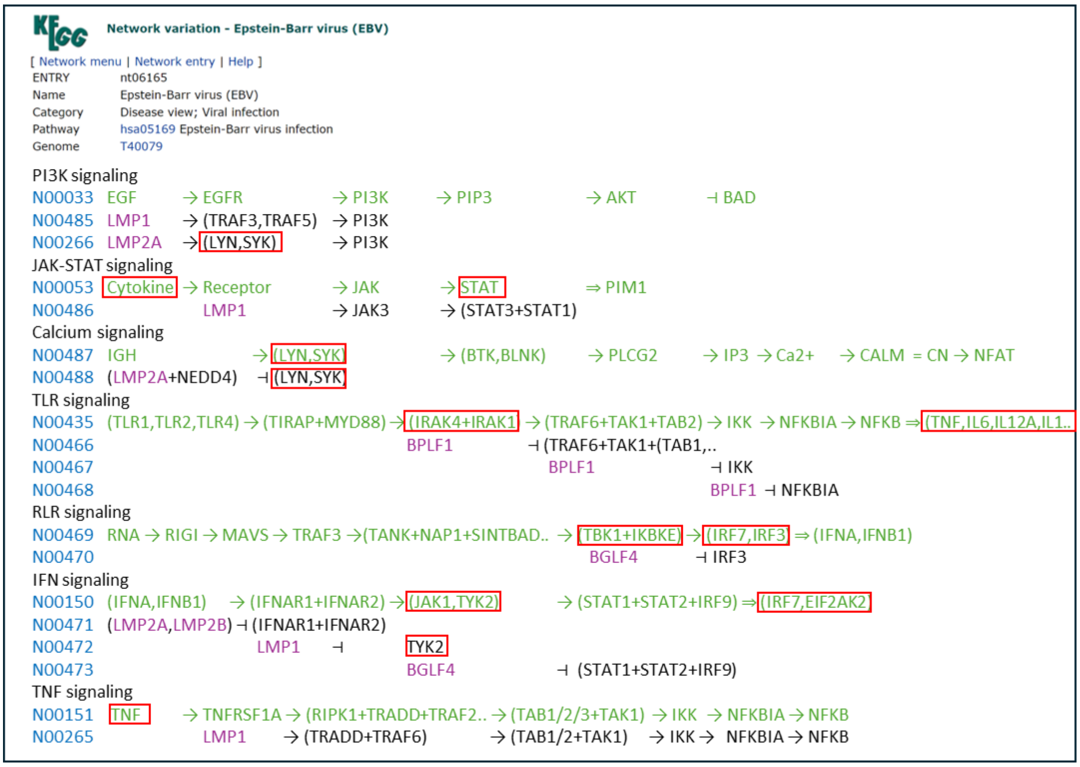

Network variation - Epstein-Barr virus (EBV)
[Network menu | Network entry | Help]
ENTRY nt06165
Name Epstein-Barr virus (EBV)
Category Disease view; Viral infection
Pathway hsa05169 Epstein-Barr virus infection
Genome T40079
PI3K signaling
N00033 EGF → EGFR → PI3K → PIP3 → AKT ⊣ BAD
N00485 LMP1 → (TRAF3,TRAF5) → PI3K
N00266 LMP2A → (LYN,SYK) → PI3K
JAK-STAT signaling
N00053 Cytokine → Receptor → JAK → STAT ⇒ PIM1
N00486 LMP1 → JAK3 → (STAT3+STAT1)
Calcium signaling
N00487 IGH → (LYN,SYK) → (BTK,BLNK) → PLCG2 → IP3 → Ca2+ → CALM = CN → NFAT
N00488 (LMP2A+NEDD4) ⊣ (LYN,SYK)
TLR signaling
N00435 (TLR1,TLR2,TLR4) → (TIRAP+MYD88) → (IRAK4+IRAK1) → (TRAF6+TAK1+TAB2) → IKK → NFKBIA → NFKB ⇒ (TNF,IL6,IL12A,IL1..
N00466 BPLF1 ⊣ (TRAF6+TAK1+(TAB1,..
N00467 BPLF1 ⊣ IKK
N00468 BPLF1 ⊣ NFKBIA
RLR signaling
N00469 RNA → RIGI → MAVS → TRAF3 → (TANK+NAP1+SINTBAD.. → (TBK1+IKBKE) → (IRF7,IRF3) ⇒ (IFNA,IFNB1)
N00470 BGLF4 ⊣ IRF3
IFN signaling
N00150 (IFNA,IFNB1) → (IFNAR1+IFNAR2) → (JAK1,TYK2) → (STAT1+STAT2+IRF9) ⇒ (IRF7,EIF2AK2)
N00471 (LMP2A,LMP2B) ⊣ (IFNAR1+IFNAR2)
N00472 LMP1 ⊣ TYK2
N00473 BGLF4 ⊣ (STAT1+STAT2+IRF9)
TNF signaling
N00151 TNF → TNFRSF1A → (RIPK1+TRADD+TRAF2.. → (TAB1/2/3+TAK1) → IKK → NFKBIA → NFKB
N00265 LMP1 → (TRADD+TRAF6) → (TAB1/2+TAK1) → IKK → NFKBIA → NFKB

Fig. S4 Risk genes of MS (**A**) and SLE (**B**) mapped to the network variation map “(nt06165) Epstein-Barr virus”. Nodes represent network elements: green, element of reference network; purple, pathogen gene/protein in variant network. Edges represent interactions/reactions: ->, activation; -|, inhibition; =>, expression. The objects mapped by KEGG Mapper tool are surrounded by red frames.

KEGG **Network variation - Parkinson disease**

[Network menu | Network entry | Help]

ENTRY	nt06463
Name	Parkinson disease
Category	Disease view; Neurodegenerative disease
Pathway	hsa05012 Parkinson disease
Disease	H00057 Parkinson disease

Calcium signaling
N00953 Glutamate → GRM1 → GNAQ → PLCB → IP3 → ITPR → Ca2+ → PKC ⊣ TRPC3 → Ca2+
N01032 PRKN* → PLCG1 → IP3 → ITPR → Ca2+
N00967 Ca2+(extracel – VGCC → Ca2+ – MCU → Ca2+(mito) ⊣ MPTP → CYCS = APAF1 → CASP9 → (CASP3,CASP7)
N01031 Ca2+(extracel– SNCA* → Ca2+ – MCU → Ca2+(mito) ⊣MPTP→ CYCS = APAF1 → CASP9 → CASP3
Ubiquitin-proteasome system
N01019 UBCH7/8 = PRKN ⊣ alphaSp22
N01020 UBCH7/8 ↛ PRKN* ↛ alphaSp22
N01021 UBCH7/8 = PRKN ⊣ PAELR
N01022 UBCH7/8 ↛ PRKN* ↛ PAELR
N01023 UBCH7/8 = PRKN ⊣ CDCREL1
N01024 UBCH7/8 ↛ PRKN* ↛ CDCREL1
N01025 UBCH7/8 = PRKN ⊣ SYPH1
N01026 UBCH7/8 ↛ PRKN* ↛ SYPH1
N01029 (Protein+UB) – 26S → Peptide
N01030 SNCA* ⊣ 26S
N01027 UB(chain) – UCHL1 → UB(free)
N01028 UB(chain) ↛ UCHL1* ↛ UB(free)
Unfolded protein response (UPR) signaling
N01009 EIF2AK3 → EIF2S1 → ATF4 ⇒ DDIT3
N01035 SNCA* ⊣ BIP ⊣ EIF2AK3 → EIF2S1 → ATF4 ⇒ DDIT3
N01011 ERN1 → XBP1 ⇒ DDIT3
N01034 SNCA* ⊣ BIP ⊣ ERN1 → XBP1
N01015 ATF6 ⇒ (DDIT3,XBP1,HSPA5)
N01033 SNCA* ⊣ BIP ⊣ ATF6 ⇒ DDIT3
Mitophagy
N01052 PINK1 → PRKN ⊣ MFN1/2
N01053 PINK1* ↛ PRKN ↛ MFN1/2
N01054 PRKN* ↛ MFN1/2
Apoptosis
N00098 (PMAIP1,BBC3,BAD,B.. ⊣ (BCL2,BCL2L1) ⊣ (BAX,BAK1) → CYCS = APAF1 → CASP9 → (CASP3,CASP7)
N01051 DJ1* ↛ BCL2L1 ↛ BAX
N01050 PINK1* ↛ PRKN ↛ BAX → CYCS = APAF1 → CASP9 → CASP3
N01049 PRKN* ↛ BAX → CYCS = APAF1 → CASP9 → CASP3
N01048 PINK1* ↛ (HTRA2,TRAP1) ↛ CYCS = APAF1 → CASP9 → CASP3
N01047 LRRK2* → CYCS = APAF1 → CASP9 → CASP3
N01056 FASLG → FAS → (DAXX+ASK1) → JNK
N01057 DJ1* ↛ (DAXX+ASK1) → JNK
N01417 PQ → ROS ⊣ TRX1 ⊣ ASK1 → JNK
Dopamine metabolism
N01530 Tyr+BH4 – TH → L -Dopa – DDC → Dopamine – MAO → DOPAL
N01037 SNCA* ↛ TH → L -Dopa
N01039 PRKN* ↛ MAO → DOPAL
N01040 Dopamine – VMAT → Dopamine(SV)
N01041 SNCA* ⊣ VMAT2
Oxidative phosphorylation
N00995 NADH – CxI → Q
N01042 SNCA* ⊣ CxI → Q
N01043 PINK1* ⊣ CxI → Q
N01044 MPP+ ⊣ CxI → Q
N01045 Rotenone ⊣ CxI → Q
N00988 Succinate – CxII → QH2
N01402 Mn2+ ⊣ CxII → QH2
N00990 QH2 – CxIII → CytC
N01046 Maneb ⊣ CxIII → CytC
Microtubule-based transport
N00976 (DCTN+DNAH+DNAI+DN.. = (TUBA+TUBB)
N01055 SNCA* ⊣ (KIF5+KLC) ↛ (TUBA+TUBB)
Transcription
N01058 DJ1* ↛ TP53 ⇒ DUSP1
N00243 (O2-,HO2,H2O2,OH,A.. ⊣ KEAP1 ⊣ NRF2 ⇒ (HMOX1,NQO1,GST,TX..
N01059 DJ1* ↛ NRF2 ↛ TRX1

Fig. S5 PD expression data mapped to the network variation map "(nt06463) Parkinson disease".

Nodes represent network elements: green, element of reference network; red, human gene variant in variant network. Edges represent interactions/reactions: ->, activation; -- (node) ->, transport through the node; -|, inhibition; -/-, missing interaction; =>, expression; =, complex formation. The objects mapped by KEGG Mapper tool are surrounded by red frames.

Supplementary Table S4: The number of patients given “medical care recipient certificate” (FY2021) in Japan (P), drugs in clinical-trial data (D), and GWAS catalog “associations” (July 2024) (G) of 338 RIDs

No.	Disease name	P	D	G
1	Spinal and bulbar muscular atrophy	1641	10	
2	Amyotrophic lateral sclerosis	9968	191	345
3	Spinal muscular atrophy	929	41	
4	Primary lateral sclerosis	140	9	
5	Progressive supranuclear palsy	12557	49	34
6	Parkinson disease	140473	377	728
7	Corticobasal degeneration	4517	18	8
8	Huntington disease	918	65	22
9	Neuroacanthocytosis	35	0	
10	Charcot-Marie-Tooth disease	781	12	11
11	Myasthenia gravis	25568	77	36
12	Congenital myasthenic syndrome	11	5	
13	Multiple sclerosis/Neuromyelitis optica	21967	424	814
14	Chronic inflammatory demyelinating polyneuropathy	5108	47	
15	Inclusion body myositis	756	19	5
16	Crow-Fukase syndrome	223	8	
17	Multiple system atrophy	11255	62	42
18	Spinocerebellar degeneration	26630	33	
19	Lysosomal storage disease	1582	116	
20	Adrenoleukodystrophy	252	30	
21	Mitochondrial disease	1608	22	
22	Moyamoya disease	13431	23	81
23	Prion disease	481	7	32
24	Subacute sclerosing panencephalitis	66	0	
25	Progressive multifocal leukoencephalopathy	88	29	
26	HTLV-1-associated myelopathy	977	33	8
27	Idiopathic basal ganglia calcification	127	0	
28	Systemic amyloidosis	4588	87	

Supplementary Table S4: (Continued)

No.	Disease name	P	D	G
29	Ullrich disease	21	0	
30	Distal myopathy	294	3	
31	Bethlem myopathy	19	0	
32	Autophagic vacuolar myopathy	9	0	
33	Schwartz-Jampel syndrome	1	0	
34	Neurofibromatosis	4056	87	2
35	Pemphigus	3236	64	15
36	Epidermolysis bullosa	290	56	
37	Generalised pustular psoriasis	2070	22	2
38	Stevens-Johnson syndrome	169	24	74
39	Toxic epidermal necrolysis	70	23	74
40	Takayasu arteritis	4587	37	166
41	Giant cell arteritis	2066	46	
42	Polyarteritis nodosa	2186	27	
43	Microscopic polyangiitis	10626	40	
44	Wegener granulomatosis	3223	42	
45	Eosinophilic granulomatosis with Polyangiitis	5839	32	
46	Malignant rheumatoid arthritis	5075	384	3277
47	Buerger disease	1858	8	
48	Primary antiphospholipid syndrome	892	2	
49	Systemic lupus erythematosus	64304	189	1534
50	Dermatomyositis	25259	63	30
51	Scleroderma	26851	200	245
52	Mixed connective tissue disease	10009	3	
53	Sjogren syndrome	18118	131	56
54	Adult still disease	4206	1	
55	Relapsing polychondritis	936	7	
56	Behcet disease	15122	48	44
57	Idiopathic dilated cardiomyopathy	18724	19	42
58	Hypertrophic cardiomyopathy	4201	60	115
59	Restricted cardiomyopathy	60	0	

Supplementary Table S4: (Continued)

No.	Disease name	P	D	G
60	Aplastic anemia	8348	97	10
61	Autoimmune hemolytic anemia	1178	47	
62	Paroxysmal nocturnal hemoglobinuria	959	39	
63	Idiopathic thrombocytopenic purpura	16972	67	
64	Thrombotic thrombocytopenic purpura	361	31	
65	Primary immunodeficiency	1964	140	
66	IgA nephropathy	12447	78	
67	Polycystic kidney disease	12164	57	
68	Ossification of the ligamentum flavum	6104	5	
69	Ossification of posterior longitudinal ligament	32406	0	8
70	Spinal stenosis	5000	96	12
71	Idiopathic osteonecrosis of the femoral head	18817	8	56
72	Pituitary ADH secretion disorder	3701	8	
73	TSH-secreting pituitary adenoma	173	2	
74	Prolactin secreting pituitary adenoma	2177	24	
75	Cushing disease	911	74	
76	Pituitary gonadotropin secretion hyperthyroidism	36	22	
77	Growth hormone secreting pituitary adenoma	4388	2	
78	Hypopituitarism	19006	61	
79	Homozygous familial hypercholesterolemia	371	30	
80	Resistance to thyroid hormone	45	2	
81	Congenital adrenal hyperplasia	945	31	
82	Congenital adrenal hypoplasia	52	0	
83	Addison disease	327	26	
84	Sarcoidosis	15655	122	182
85	Idiopathic interstitial pneumonia	17665	145	
86	Pulmonary arterial hypertension	4319	147	14
87	Pulmonary veno-occlusive disease	24	4	
88	Chronic thromboembolic pulmonary hypertension	4843	38	
89	Lymphangioleiomyomatosis	912	28	1
90	Retinitis pigmentosa	22223	64	31

Supplementary Table S4: (Continued)

No.	Disease name	P	D	G
91	Budd-Chiari syndrome	218	4	
92	Idiopathic portal hypertension	301	0	
93	Primary biliary cholangitis	16996	74	
94	Primary sclerosing cholangitis	1022	42	
95	Autoimmune hepatitis	6884	50	16
96	Crohn disease	48320	274	1092
97	Ulcerative colitis	138079	241	801
98	Eosinophilic gastrointestinal disease	1184	64	
99	Chronic intestinal pseudo-obstruction	186	3	
100	Megacystis microcolon intestinal hypoperistalsis syndrome	1	0	
101	Congenital isolated hypoganglionosis	17	0	
102	Rubinstein-Taybi syndrome	7	1	
103	Cardio-facio-cutaneous syndrome	7	0	
104	Costello syndrome	10	0	
105	CHARGE syndrome	25	0	
106	Cryopyrin-associated periodic syndrome	83	5	
107	Juvenile idiopathic arthritis	918	74	178
108	TNF receptor-associated periodic syndrome	33	1	
109	Atypical hemolytic uremic syndrome	78	7	
110	Blau syndrome	22	0	
111	Congenital myopathy	351	5	
112	Marinesco-Sjogren syndrome	5	0	
113	Muscular dystrophy	5246	122	47
114	Non-dystrophic myotonia syndrome	23	7	
115	Hereditary periodic paralysis	60	2	
116	Atopic myelitis	47	0	
117	Syringomyelia	602	1	
118	Myelomeningocele	124	8	
119	Isaacs syndrome	108	0	
120	Hereditary dystonia	113	4	
121	Neuroferritinopathy	2	0	

Supplementary Table S4: (Continued)

No.	Disease name	P	D	G
122	Superficial siderosis	198	3	
123	Cerebral autosomal recessive arteriopathy with subcortical infarcts and leukoence…	5	0	
124	Cerebral autosomal dominant arteriopathy with subcortical infarcts and leukoence..	187	10	
125	Hereditary diffuse leukoencephalopathy with spheroid	65	1	
126	Perry syndrome	5	0	
127	Frontotemporal lobar degeneration	1311	44	
128	Bickerstaff brainstem encephalitis	77	0	
129	Acute encephalopathy with biphasic seizures and late reduced diffusion	46	2	2
130	Congenital insensitivity to pain with anhydrosis	42	0	
131	Alexander disease	46	0	
132	Congenital supranuclear bulbar palsy	8	0	
133	Moebius syndrome	13	0	
134	Septo-optic dysplasia	13	0	
135	Aicardi syndrome	15	3	
136	Hemimegalencephaly	24	0	
137	Focal cortical dysplasia	72	2	
138	Nerve cell migration disorder	60	0	
139	Congenital cerebral hypomyelination	38	1	
140	Dorabe syndrome	67	12	
141	Mesial temporal lobe epilepsy with hippocampal sclerosis	73	0	2
142	Myoclonic absence epilepsy	4	0	
143	Epilepsy with myoclonic-atonic seizure	22	0	
144	Lennox-Gastaut syndrome	282	14	
145	West syndrome	229	30	
146	Ohtahara syndrome	20	0	
147	Early myoclonic encephalopathy	10	0	
148	Epilepsy of infancy with migrating focal seizure	21	0	
149	Hemiconvulsion hemiplegia epilepsy syndrome	33	12	
150	Ring chromosome 20 epilepsy syndrome	15	0	
151	Rasmussen encephalitis	46	3	
152	PCDH19 related syndrome	13	1	

Supplementary Table S4: (Continued)

No.	Disease name	P	D	G
153	Acute encephalitis with refractory, repetitive partial seizure	55	0	
154	Epilepsy with continuous spikes and waves during slow sleep	27	6	
155	Acquired aphasia with convulsive disorder	8	3	
156	Rett syndrome	98	27	
157	Sturge-Weber syndrome	77	7	
158	Tuberous sclerosis	925	32	
159	Xeroderma pigmentosum	86	4	
160	Congenital ichthyosis	90	22	
161	Familial benign chronic pemphigus	57	10	
162	Pemphigoid	3764	81	
163	Idiopathic pure sudomotor failure	551	0	
164	Oculocutaneous albinism	28	29	
165	Pachydermoperiostosis	19	0	
166	Pseudoxanthoma elasticum	109	13	
167	Marfan syndrome	1081	19	
168	Ehlers-Danlos syndrome	179	17	
169	Menkes disease	1	2	
170	Occipital horn syndrome	1	2	
171	Wilson disease	715	14	
172	Hypophosphatasia	26	4	
173	VATER syndrome	15	0	
174	Nasu-Hakola disease	4	0	
175	Weaver syndrome	0	0	
176	Coffin-Lowry syndrome	5	0	
177	Joubert syndrome related disorder	11	0	
178	Mowat-Wilson syndrome	13	0	
179	Williams syndrome	49	9	
180	ATR-X syndrome	7	0	
181	Crouzon syndrome	19	0	
182	Apert syndrome	6	0	
183	Pfeiffer syndrome	6	0	

Supplementary Table S4: (Continued)

No.	Disease name	P	D	G
184	Antley-Bixler syndrome	3	0	
185	Coffin-Siris syndrome	4	0	
186	Rothmund-Thomson syndrome	3	1	
187	Kabuki syndrome	14	3	
188	Polysplenia syndrome	54	0	
189	Asplenia syndrome	84	0	
190	Branchio-oto-renal syndrome	8	0	
191	Werner syndrome	102	2	
192	Cockayne syndrome	5	3	
193	Prader-Willi syndrome	172	28	
194	Sotos syndrome	17	0	
195	Noonan syndrome	45	4	
196	Young-Simpson syndrome	0	0	
197	1p36 deletion syndrome	8	0	
198	4p deletion syndrome	7	0	
199	5p deletion syndrome	6	0	
200	Paternal uniparental disomy of chromosome 14	6	0	
201	Angelman syndrome	30	9	
202	Smith-Magenis syndrome	1	4	
203	22q11.2 deletion syndrome	69	1	
204	Emanuel syndrome	5	0	
205	Fragile X syndrome related disease	6	8	
206	Fragile X syndrome	2	31	1
207	Persistent truncus arteriosus	32	0	
208	Corrected transposition of great arteries	186	0	
209	Complete transposition of great vessel	244	0	
210	Single Ventricle	425	37	
211	Hypoplastic left heart syndrome	54	16	
212	Tricuspid atresia	183	9	
213	Pulmonary atresia without ventricular septum defect	140	0	
214	Pulmonary atresia with ventricular septum defect	108	2	

Supplementary Table S4: (Continued)

No.	Disease name	P	D	G
215	Tetralogy of Fallot	689	31	6
216	Double outlet right ventricle	256	0	
217	Ebstein disease	130	0	
218	Alport syndrome	223	10	
219	Galloway-Mowat syndrome	1	0	
220	Rapidly progressive glomerulonephritis	1147	8	
221	Anti-glomerular basement membrane disease	343	1	
222	Primary nephrotic syndrome	12221	95	
223	Primary membranoproliferative glomerulonephritis	367	0	
224	Purpura nephritis	1023	35	
225	Congenital nephrogenic diabetes insipidus	45	32	
226	Interstitial cystitis with Hunners ulcer	885	74	
227	Osler disease	837	36	
228	Bronchiolitis obliterans	35	43	
229	Autoimmune pulmonary alveolar proteinosis	212	12	2
230	Alveolar hypoventilation syndrome	145	7	
231	Alpha-1-antitrypsin deficiency	16	21	
232	Carney complex	23	3	
233	Wolfram syndrome	12	9	
234	Peroxisomal disease (except Adrenoleukodystrophy)	0	12	
235	Hypoparathyroidism	302	43	
236	Pseudohypoparathyroidism	116	6	
237	ACTH unresponsiveness	14	0	
238	Vitamin D-resistant rickets	396	13	
239	Vitamin D-dependent rickets	5	0	
240	Phenylketonuria	264	12	
241	Hypertyrosinemia type I	2	1	
242	Hypertyrosinemia type II	0	0	
243	Hypertyrosinemia type III	1	0	
244	Maple syrup urine disease	13	0	
245	Propionic acidemia	15	4	

Supplementary Table S4: (Continued)

No.	Disease name	P	D	G
246	Methylmalonic acidemia	30	4	
247	Isovaleric acidemia	3	0	
248	Glucose transporter type 1 deficiency	14	1	
249	Glutaric acidemia type 1	4	0	
250	Glutaric acidemia type 2	7	0	
251	Urea cycle disorder	92	16	
252	Lysinuric protein intolerance	27	0	
253	Congenital folate malabsorption	0	0	
254	Porphyria	47	26	
255	Multiple carboxylase deficiency	6	0	
256	Muscle glycogenosis	22	30	
257	Hepatic glycogenosis	101	16	
258	Galactose-1-phosphate uridylyltransferase deficiency	2	0	
259	Lecithin-cholesterol acyltransferase deficiency	4	1	
260	Sitosterolemia	24	6	
261	Tangier disease	9	1	
262	Primary hyperchylomicronemia	46	0	
263	Cerebrotendinous xanthomatosis	48	2	
264	Abetalipoproteinemia	4	0	
265	Lipodystrophy	33	69	
266	Familial mediterranean fever	530	6	23
267	Hyper-IgD syndrome	2	1	
268	Nakajo-Nishimura syndrome	7	2	
269	Pyogenic arthritis	7	20	
270	Chronic recurrent multifocal osteomyelitis	102	12	
271	Ankylosing spondylitis	4552	98	412
272	Fibrodysplasia ossificans progressiva	25	13	
273	Congenital scoliosis with rib anomaly	21	0	
274	Osteogenesis Imperfecta	121	22	
275	Thanatophoric dysplasia	5	0	
276	Achondroplasia	88	10	

Supplementary Table S4: (Continued)

No.	Disease name	P	D	G
277	Lymphangiomatosis	56	2	
278	Huge lymphatic malformation with cervicofacial lesion	17	10	
279	Huge venous malformation with cervical, oral and pharyngeal diffuse lesion	47	12	
280	Huge arteriovenous malformation with cervicofacial or limb lesion	104	30	
281	Klippel-Trenaunay-Weber syndrome	239	1	
282	Congenital dyserythropoietic anemia	11	4	
283	Acquired pure red cell aplasia	829	28	
284	Diamond-Blackfan anemia	23	44	
285	Fanconi anemia	13	41	
286	Hereditary sideroblastic anemia	13	14	
287	Epstein syndrome	12	0	
288	Autoimmune acquired coagulation factor deficiency	414	24	
289	Cronkhite-Canada syndrome	189	0	
290	Chronic nonspecific multiple ulcers of the small intestine	88	1	
291	Hirschsprung disease, entire colon type	19	19	
292	Cloacal exstrophy	16	0	
293	Persistent cloaca	42	0	
294	Congenital diaphragmatic hernia	13	11	
295	Infant huge hepatic hemangioma	0	0	
296	Biliary atresia	422	55	41
297	Alagille syndrome	41	7	2
298	Hereditary pancreatitis	31	75	
299	Cystic fibrosis	12	299	36
300	IgG4-related disease	3371	38	
301	Macular dystrophy	217	9	
302	Leber hereditary optic neuropathy	126	5	
303	Usher syndrome	20	1	
304	Juvenile-onset bilateral sensorineural hearing loss	42	0	
305	Delayed endolymphatic hydrops	27	2	
306	Eosinophilic sinusitis	17525	0	
307	Canavan disease	1	10	

Supplementary Table S4: (Continued)

No.	Disease name	P	D	G
308	Progressive leukoencephalopathy	20	0	
309	Progressive myoclonus epilepsy	41	4	
310	Congenital anomalies syndrome	30	8	
311	Congenital tricuspid stenosis	3	0	
312	Congenital mitral stenosis	12	0	
313	Congenital pulmonary vein stenosis	1	0	
314	Vascular sling	2	0	
315	Nail-Patella syndrome	7	0	
316	Carnitine cycle disorder	19	9	
317	Trifunctional protein deficiency	4	5	
318	Citrin deficiency	62	0	
319	Sepiapterin reductase deficiency	2	0	
320	Inherited glycosylphosphatidylinositol deficiency	0	4	
321	Non-ketotic hyperglycinemia	2	1	
322	Beta-ketothiolase deficiency	0	0	
323	Aromatic L-amino acid decarboxylase deficiency	4	2	
324	Methylglutaconic aciduria	1	3	
325	Hereditary autoinflammatory syndrome	13	8	
326	Osteopetrosis	23	13	
327	Idiopathic thrombosis	205	0	
328	Anterior segment dysgenesis	13	0	
329	Aniridia	120	2	
330	Congenital tracheal stenosis	41	1	
331	Idiopathic multicentric castleman disease	1526	26	
332	Gelatinous drop-like corneal dystrophy	4	0	
333	Hutchinson-Gilford syndrome	0	6	
334	Cerebral creatine deficiency syndrome	0	0	
335	Nephronophthisis	3	0	
336	Familial hypobetalipoproteinemia 1	0	0	
337	Homocystinuria	11	5	
338	Progressive familial intrahepatic cholestasis	2	10	
